# Effects of annulus defects and implantation of poly(lactic-co-glycolic acid) (PLGA)/fibrin gel scaffolds on nerves ingrowth in a rabbit model of annular injury disc degeneration

**DOI:** 10.1186/s13018-017-0572-5

**Published:** 2017-05-12

**Authors:** Long Xin, Weixing Xu, Leijun Yu, Shunwu Fan, Wei Wang, Fang Yu, Zhenbin Wang

**Affiliations:** 1 Department of Spine Surgery, Tongde Hospital of Zhejiang Province, Hangzhou, China; 2 Department of Orthopedics, the Affiliated Sir Run Run Shaw Hospital, Zhejiang University, Zhejiang, China; 3 Department of Polymer Materials Science and Engineering, School of Material Science and Engineering, Tianjin University, Tianjin, China; 4 Orthopedics Laboratory, Department of Spine Surgery, The Fourth Affiliated Hospital, Xinjiang Medical University, Urumqi, Xinjiang 830000 China; 5 Department of Mental Health, Tongde Hospital of Zhejiang Province, Hangzhou, China

**Keywords:** PLGA scaffold, Fibrin gel, Proteoglycan, Nerve ingrowth, Blood vessels, Annular injury model

## Abstract

**Background:**

Growth of nerve fibers has been shown to occur in a rabbit model of intravertebral disc degeneration (IVD) induced by needle puncture. As nerve growth may underlie the process of chronic pain in humans affected by disc degeneration, we sought to investigate the factors underlying nerve ingrowth in a minimally invasive annulotomy rabbit model of IVD by comparing the effects of empty disc defects with those of defects filled with poly(lactic-co-glycolic acid)/fibrin gel (PLGA) plugs.

**Methods:**

New Zealand white rabbits (*n* = 24) received annular injuries at three lumbar levels (L3/4, L4/5, and L5/6). The discs were randomly assigned to four groups: (a) annular defect (1.8-mm diameter; 4-mm depth) by mini-trephine, (b) annular defect implanted with a PLGA scaffold containing a fibrin gel, (c) annular puncture by a 16G needle (5-mm depth), and (d) uninjured L2/3 disc (control). Disc degeneration was evaluated by radiography, MRI, histology, real-time PCR, and analysis of proteoglycan (PG) content. Nerve ingrowth into the discs was assessed by immunostaining with the nerve marker protein gene product 9.5.

**Results:**

Injured discs showed a progressive disc space narrowing with significant disc degeneration and proteoglycan loss, as confirmed by imaging results, molecular and compositional analysis, and histological examinations. In 16G punctured discs, nerve ingrowth was observed on the surface of scar tissue. In annular defects, nerve fibers were found to be distributed along small fissures within the fibrocartilaginous-like tissue that filled the AF. In discs filled with PLGA/ fibrin gel, more nerve fibers were observed growing deeper into the inner AF along the open annular track.  In addition, innervations scores showed significantly higher than those of punctured discs and empty defects. A limited vascular proliferation was found in the injured sites and regenerated tissues.

**Conclusions:**

Nerve ingrowth was significantly higher in PLGA/fibrin-filled discs than in empty defects. Possible explanations include (i) annular fissures along the defect and early loss of proteoglycan may facilitate the ingrowth process and (ii) biodegradable PLGA/fibrin gel may promote adverse growth of nerves and blood vessels into deeper parts of injured disc. The rabbit annular defect model of disc degeneration appears suitable to investigate the effects of nerve ingrowth in relation to pain generation.

## Background

Low back pain is a common musculoskeletal problem in orthopedics, particularly in the case of degeneration of the intervertebral disc (IVD) [1–3]. The degenerative processes frequently result in annular rupture [4, 5], which ultimately results in the reduction of cell numbers in the nucleus pulposus (NP), altered collagen type, loss of proteoglycans, and increase of catabolic activity [6–8]. At the same time, innervated and vascularized tissue progressively invades the annular fissure, which may provide a chemically and mechanically favorable environment for perivascular nerve growth [9–13]. The presence of nerve fibers in the inner layers of the annulus fibrosus (AF) and NP, in turn, is a potential source of pain in patients affected by IVD [11, 12, 14, 15].

Rabbit disc models have been developed for the study of IVD degeneration and experimental interventions, due to acceptable similarities with the pathological process in humans [9, 16, 17]. In particular, the presence of nerve fibers was only observed in the scar tissue produced by needle puncture, possibly resulting from instantaneous plugging of the track lesion with jelly-like NP, meant in turn to prevent an invasion by scar-like tissues [9]. In addition, the extruded NP was surrounded by scar tissue, which rather tends to be spontaneously resorbed in human beings [18]. Elegant studies have shown that aggrecan derived from both the AF and NP has inhibitory effect on nerve ingrowth into the IVD [10, 19, 20]. Also, loss of glycosaminoglycans (GAG) in annular lesion models of IVD may be associated with more extensive AF structural defects than needle puncture injury [21, 22].

To date, there have been only limited investigations on the nerve ingrowth in models of IVD degeneration in the rabbit. A minimally invasive annulotomy-induced rabbit model was successfully established in our group, targeted at the repair of the AF by implantation of PLGA poly(lactic-co-glycolic acid) [23]. Briefly, open mini-trephine injuries of the AF resulted in a slow repair of the annular wall, minimizing the risk of early sealing due to the extruded NP surrounded by scar tissue in the injured site. During the repair process, the porous PLGA scaffold favors cell or tissue ingrowth by providing a structural support. Recent studies have shown that PLGA constructs are highly effective also in promoting tissue repair, including the nerves [24–26] and blood vessels [27–29]. Also, fibrin hydrogels are routinely used for surgical hemostasis and tissue adhesion [30, 31]. In the present study, we hypothesized that (i) annular fissure within the open annular defect may be associated with the ingrowth of nerves, (ii) PLGA-coated fibrin gel porous scaffold may promote peripheral nerves ingrowth into deeper parts of degenerated discs, and (iii) early loss of proteoglycan may facilitate nerve growth. For this purpose, several morphological parameters were assessed in the model of rabbit annular injury degeneration, in particular by comparing the characteristics of defects filled with porous PLGA scaffold with those of two types of empty defects (trephine- and needle-induced).

## Methods

### Preparation of PLGA-coated fibrin gel constructs

The PLGA sponges were fabricated by a porogen-leaching method using gelatin spheres with a size of 280–450 μm as reported previously [32]. The PLGA sponges were made into plugs of 1.8-mm diameter and 4-mm length. The plugs were sterilized by ethylene oxide. The fibrinogen was isolated from fresh human plasma (Blood Center of Zhejiang Province of China) by a freezing-thawing cycle [33]. Briefly, the fresh human plasma was frozen at 20 °C for 24 h and then thawed at 4 °C for 18 h. Thereafter, the plasma was centrifuged at 6500×*g* for 20 min. The precipitate was dissolved in 0.9% NaCl solution, which was then frozen at 20 °C for 2 h and lyophilized for 18 h to obtain the fibrinogen. The fibrinogen (40 mg/mL, 0.9% NaCl solution) and thrombin (Sigma, 5 U/mL in 40 mM CaCl2 solution) solutions were sterilized by filtering through syringe filters. The final fibrinogen concentration used in all the experiments was 20 mg/mL, as previously described [30]. The porous PLGA plugs were immersed into the homogeneous fibrinogen solution under reduced pressure. The composite scaffolds were then lyophilized overnight to dry and subsequently stored at −20 °C until further use.

### Animal surgery

**Fig. 1:**
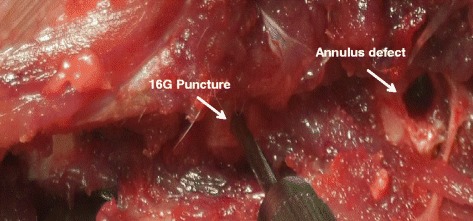
Surgical technique. Exposure of three consecutive intervertebral discs (L3/4, L4/5, and L5/6). Empty defects (1.8-mm diameter; 4-mm depth) were created using a mini-trephine in the anterolateral annulus; annulus punctures were performed using a 16G needle at a depth of 5 mm

A total of 24 New Zealand rabbits (age 8.21 ± 1.16 months, weight 3.24 ± 0.21 kg) were supplied by the Laboratory Animal Center of Zhejiang province. Protocols were conducted in accordance with the Guidance for the Care and Use of Laboratory Animals, as formulated by the Ministry of Science and Technology of the People’s Republic of China, and the “Principles of laboratory animal care” (NIH publication No. 86-23, revised 1985) were followed. The rabbits were randomly allocated to 0.5-, 1-, 3-, and 6-month survival groups (*n* = 6 in each group). The surgical procedures of implantation PLGA was used as described previously [23]. An anterolateral retroperitoneal approach was used to expose three consecutive levels of the rabbit IVD, comprising L3/4, L4/5, and L5/6 (Fig. 1). Annular injuries were randomly allocated to four disc levels: (1) AF defect group: annular defects (1.8-mm diameter; 4-mm depth) were created using a mini-trephine, (2) implantation group: annular defects were filled with a PLGA/fibrin gel plug, (3) puncture group: annular puncture was created by 16G needle at a depth of 5 mm, as previously described [9, 34, 35], and (4) intact group: the L2/3 disc served as uninjured control. Finally, the wound was closed in layers. Following surgery, the rabbits were permitted free cage activity and food and water ad libitum.

### Radiographic analyses and magnetic resonance imaging

**Fig. 2:**
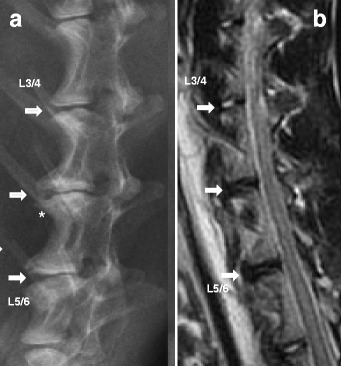
Representative lateral radiographs and magnetic resonance image (MRI) of intervertebral discs at 6 months after surgery. **a** The experimental segments showed obvious and various degrees of disc space narrowing. Osteophyte formation (*white asterisk*) was evident at the margin of the vertebral bodies. **b** Significant low T2-weighted signal intensity was clearly visible in the experimental segments after surgery. The *arrows* indicate the operated disc

Lateral plain radiographs were performed under general anesthesia (sodium pentobarbital, 30 mg/kg) at 0.5, 1, 3, and 6 months after surgery (*n* = 6 per time point). Lateral radiographs were obtained using a DR machine (General Electric Healthcare, Bucks, Great Britain) (Fig. 2a). Analysis of disc height was performed as previously described [34, 36]. The mean disc height index (DHI) was the ratio of the average measurements obtained from the anterior, middle, and posterior portions of the IVD and the average of adjacent vertebral body heights. Changes in the DHI were expressed as DHI% and normalized to the measured preoperative DHI (DHI% = postoperative DHI/preoperative DHI × 100). All measurements were done using the picture archiving and communication system (PACS) routinely used in the local hospital. At 1 and 6 months after surgery (*n* = 6), MRI examinations were performed using a 1.5-T Imager with a quadrature extremity coil receiver (Fig. 2b). Midsagittal T2-weighted images were obtained in the following settings: fast spin echo sequence with time to repetition (TR) of 3500 ms, time to echo (TE) of 100 ms, 320(h) × 256(v) matrix; field of view of 260; number of excitations of 4; and slice thickness of 2 mm with a 0-mm gap. The MRI scans were evaluated by two blinded observers using the Pfirrmann’s classification scores [37] based on the changes of degree and area of signal intensity.

### Tissue harvesting

At 1 and 6 months after surgery (*n* = 6), the rabbits were euthanized by intravenous sodium pentobarbital overdose for histology and immunohistology analysis. The experimental IVDs (L2/3, L3/4, L4/5, and L5/6), including approximately one-third of the adjacent vertebral bodies, were harvested from each of the lumbar spine under sterile conditions. The specimens were then dissected sagittally and divided into two symmetric parts. From one-half of each disc, the NP was carefully separated from the AF and then snap-frozen in liquid nitrogen, with subsequent storage at −80 °C in preparation for PCR analysis. The other half was used for histological analysis.

### Sulfated glycosaminoglycan content measurement

The entire jelly-like NP was isolated from each level discs at 6 months after surgery (*n* = 6). The amount of proteoglycan content, mainly s-GAG, was quantified using the 1,9-dimethylmethylene blue (DMMB) method [38, 39]. Briefly, each lyophilized sample was digested with 125 μg/mL papain (Sangon Inc, ShangHai; PRC) in sterile PBS, 5 mM EDTA, and 5 mM cysteine · HCl at pH 6.8 and 60 °C overnight. After complete digestion, 20 μL papain digest were added to 200 μL of DMMB solution, with absorbance detected at 520 nm. Total sGAG in the disc for each group was normalized according to the tested DNA amount, and then the sGAG/DNA ratio was measured and reported.

### qRT-PCR analysis

Total RNA was extracted from the pulverized NP tissues using TRIzol reagent (Invitrogen, Carlsbad, CA, USA) and purified using the RNeasy Mini Kit (Qiagen Inc). After extraction, RNA was quantified using a NanoDrop N-1000 spectrophotometer (Thermo Fisher Scientific, Wilmington, DE, USA). One microgram of total RNA was reverse-transcribed into cDNA using the Superscript™ First Strand cDNA synthesis kit (Invitrogen, Carlsbad, CA, USA). The gene expression of aggrecan, type I collagen (Col1A1), type II collagen (Col2A1), MMP-3, and glyceraldehyde-3-phosphate dehydrogenase (GAPDH) in the intervertebral discs were analyzed by quantitative real-time PCR using Real-Time Detection System (Bio-Rad, Hercules, CA, USA). All primer sequences are listed in Table 1. A positive standard curve for each primer was obtained using serially diluted cDNA sample mixture. Quantifications of gene expression for aggrecan, Col1A1, Col2A1, and MMP-3 were calculated using standard curves and normalized to GAPDH in each sample, and then the expression of treated discs was normalized to control discs.

**Table 1:** Oligonucleotide primers for PCR amplification

Gene	Primer sequence(5′-3′)	Annealing temperature (°C)
GAPDH	Forward: ACTCTGGCAAAGTGGATG Reverse: TCCTGGAAGATGGTGATG	60
Aggrecan	Forward: GAGGTCGTGGTGAAAGGTGT Reverse: GTGTGGATGGGGTACCTGAC	62
COL1A1	Forward: AGGGCCAAGACGAAGACATC Reverse: AGATCACGTCATCGCACAACA	60
COL2A1	Forward: GGATAGACCCCAACCAAGGC Reverse: GCTGCTCCACCAGTTCTTCT	62
MMP-3	Forward: GCCAAGAGATGCTGTTGATG Reverse: AGGTCTGTGAAGGCGTTGTA	65

### Histology and immunohistochemistry

The specimens were fixed in 10% formalin, decalcified in ethylenediamine tetraacetic acid (EDTA), and processed for paraffin sectioning. Blocks of tissue were embedded in paraffin and sliced into 5-μm sections using a microtome. Sections of IVD samples were deparaffinized and stained with hematoxylin/eosin (HE) to observe changes in the AF and adjacent tissue, or with safranin-O staining for the assessment of the proteoglycan content. Alternatively, the sections were subjected to immunohistology for the nerve marker protein gene product 9.5 (PGP9.5). All stained sections were analyzed under an optical microscope (Leica Microscope, Wetzlar, Germany) at magnifications ranging from ×40 to ×400.

To perform immunohistological staining for PGP9.5, the epitopes in the sections were first heat-induced retrieved. The sections were then subjected to blocking of the endogenous peroxidase activity with 0.5% hydrogen peroxide, blocking with 25% normal bovine serum (BSA)/tris-buffered saline for 2 h at room temperature, and overnight incubation at 4 °C with a primary antibody to mouse monoclonal antibody against human protein gene product 9.5 (diluted 1:80, Abcam, Cambridge, GB). Bound primary antibodies were then detected by incubation with a secondary antibody (anti-mouse goat IgG, dilution 1:200; MP Biomedicals, Santa Ana, CA, USA) coupled to horseradish peroxidase (HRP) for 1 h at RT and subsequent visualization of the HRP with DAB (Sigma-Aldrich, St. Louis, Missouri, USA). Thereafter, the sections were counterstained with hematoxylin, mounted with mounting medium (Sigma-Aldrich), and examined by light microscopy. As an additional control procedure, the primary antibody was omitted or replaced with non-immunoreactive sera. Control stainings yielded negative results. The presence of immunoreactive nerve fibers in the specimen was thoroughly assessed, as well as the scar tissue that formed on the surface of injured discs. The degree of ingrowth of immunoreative fibers was graded according to the method of Aoki et al [9] with a slight modification: 0 = no fibers, 1 = 1 or 2 nerves/field, 2 = nerves extending into the superficial site of the repair tissue or outer AF (>3 nerves/field), 3 = small well-defined nerves extending into as far as the newly formed tissue of inner AF, and 4 = nerves extending into the NP.

### Statistical analysis

Data were expressed as means ± standard error of the mean. Statistical analysis was performed using SPSS 18.0 software (SPSS Inc., Chicago, IL, USA). Significant differences in the radiograph measurements were analyzed by repeated measurement analysis of variance (ANOVA) and Fisher’s least significant difference (LSD) test. The effect of time after surgery was analyzed with the Kruskal-Wallis test. Mann-Whitney *U* tests were used to analyze the MRI score, innervation, gene expression, and biochemical data. The level of significance was set at *P* < 0.05.

## Results

### Characteristics of PLGA-coated fibrin gel scaffold

**Fig. 3:**
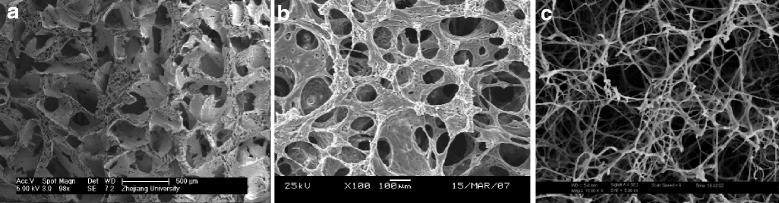
Scanning electron micrographs of PLGA sponge (**a**) and PLGA/fibrin gel constructs (**b**–**c**) at different magnification

At scanning electron microscopy, the fabricated PLGA scaffolds showed interconnected micropores with an average pore size of 350 μm (Fig. 3a). Thus, the structure of the pores was assessed as a suitable environment for tissue ingrowth. Under reduced pressure, the fibrinogen solution was well infiltrated into the pores of the sponge and the lyophilized fibrin gel was homogenously coated in the PLGA pore walls (Fig. 3b, c).

### Radiographic and MRI assessments

**Fig. 4:**
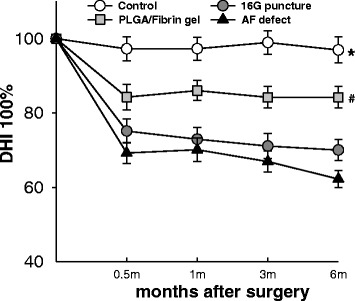
Changes in disc height index (DHI) after surgery. The operated discs showed a slow, progressive decrease in disc height over time. The DHI in the 16G puncture and annulus defect groups were significantly lower than in the control group (*P < 0.01 vs. control group). The DHI of the PLGA/fibrin gel implantation group was significantly higher than that of the 16G puncture group and annulus defect group over 6 months (^#^ *P* < 0.05 vs. 16G puncture group or annulus defect group)

Compared with normal controls, there was a slow, progressive decrease of disc height in the operated discs, and this was sustained over 6 months. The disc height index (DHI) in the 16G puncture and AF defect group decreased markedly at 0.5 months after surgery (and subsequently at a slower rate) and was significantly lower than that in the control group (*P* < 0.01). Notably, the DHI of the PLGA/fibrin gel implantation group (from now on PLGA/fibrin for brevity) was significantly higher than those in the 16G puncture group and AF defect group at 1 month post-surgery and thereafter (*P* < 0.05), whereas no significant difference was observed between 16G puncture and empty AF defect group (*P* > 0.05) (Fig. 4).

**Fig. 5:**
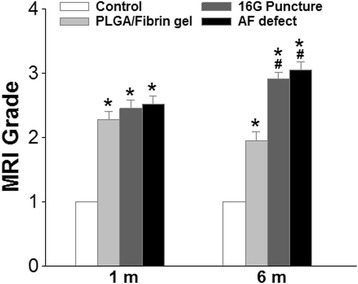
Changes in magnetic resonance imaging (MRI) after surgery. The MRI scores of the three experimental groups were significantly higher than those of the control group at 1 and 6 months (*P < 0.01 vs. control group). At 6 months, the MRI score of the PLGA/fibrin gel implantation group was lower than that of the 16G puncture group or annulus trephination (^#^ *P* < 0.05 vs. 16G puncture group or annulus defect group)

The MRI scores of the operated discs were significantly higher than those of the control discs over the whole follow-up period (1 and 6 months, *P* < 0.01). At 1 month after surgery, the MRI scores of the PLGA/fibrin group did not significantly differ from those of the 16G puncture group or AF defect group; however, significant differences became evident at 6 months (*P* < 0.05), i.e., the PLGA/fibrin group showed a significantly lower MRI score compared with either the 16G puncture group or the AF defect group (*P* < 0.05) (Fig. 5).

### Real-time PCR analysis of gene expression

**Fig. 6:**
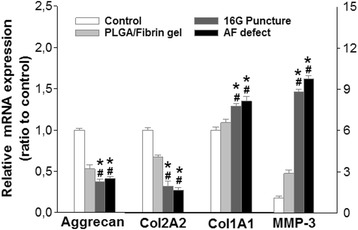
mRNA expression of aggrecan, Col1A1, Col2A1, and MMP-3 at 6 months after surgery. There was a marked reduction of aggrecan and Col2A1 and a significant increase of Col1A1 and MMP-3 expression in the injured discs, compared with those in the control group. The mRNA expression in the PLGA/fibrin gel implantation group was comparable to that in the control group (*P* > 0.05). (*P < 0.01 vs. control group; ^#^ *P* < 0.01 vs. PLGA/fibrin gel implantation group)

When compared with the control and PLGA/fibrin group, the expression of aggrecan and Col2A1 was significantly decreased, and the expression of Col1A1 and MMP-3 was markedly upregulated in both the 16G puncture group and AF defect group at 6 months after surgery (*P* < 0.01). There were no significant differences in the expression of aggrecan, Col1A1, Col2A1, and MMP-3 between the 16G puncture group and AF defect group (*P* > 0.05) (Fig. 6).

### Sulfated glycosaminoglycan content

**Fig. 7:**
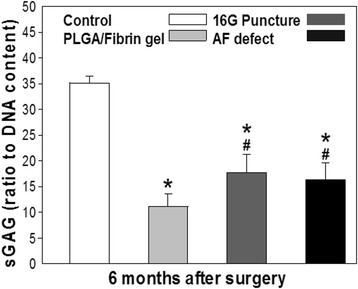
Changes in proteoglycan (sulfated glycosaminoglycan; sGAG) content of the nucleus pulposus (NP) at 6 months after surgery. IVD injury led to a decrease in proteoglycan content in the NP compared to that in control discs (*P < 0.01 vs. control group). The PLGA/fibrin gel group showed a significant decrease of the sGAG/DNA ratio compared with the 16G puncture or AF defect groups (^#^ *P* < 0.05 vs. the 16G puncture group or annulus defect group)

At 6 months post-surgery, all IVD injuries led to a decrease of proteoglycan content in the NP compared to control discs (*P* < 0.01). The decrease of sGAG/DNA ratio observed in the PLGA/fibrin group was significantly more pronounced than in the 16G puncture or AF defect groups (*P* < 0.05), whereas no significant differences were observed between the 16 G puncture and the AF defect group (*P* > 0.05) (Fig. 7).

### Histologic assessment

**Fig. 8:**
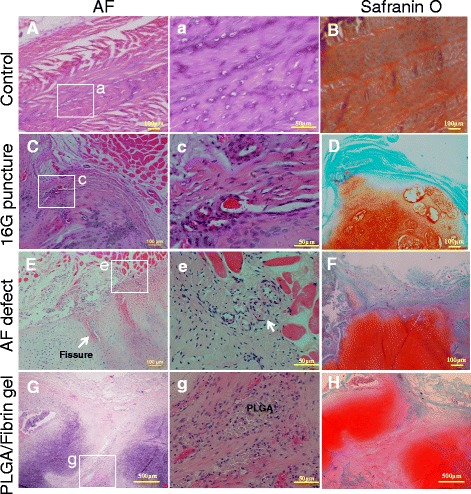
Hematoxylin/eosin (A, C, E, and G; a, c, e, and g) and safranin-O (B, D, F, and H) staining of the annulus fibrosus (AF) at 6 months after surgery. The intact AF displayed a multi-lamellar structure rich in proteoglycans, as shown by strong safranin-O staining (A, B, (a): different magnification). A disorganized, extensive scar tissue with abundant safranin-O staining was evident at the puncture site (C, D). Small blood vessels were variably evident in the outer AF (c). The annular defect was replenished by pronounced fibrocartilaginous tissue rich in proteoglycans. Small fissures were discernible as penetrations into the repair tissue at a limited depth (E, F). Blood vessels were present in the layer between the periannular muscle and the outer AF (e). Clusters of newly regenerated tissue formed a concave channel along the track of the AF defect (G) and were well integrated with the inner part of the AF (g). The proteoglycan content was markedly reduced in the AF defect track, except for a strongly safranin-O-stained region at the sites of cartilaginous tissue (H). *White boxes* indicate areas of higher magnification

At 6 months after surgery, HE staining showed that the intact AF (control group) was characteristically well organized with its multilamellar structure. The safranin-O staining indicated the presence of proteoglycan-rich matrix in the annulus (Fig. 8A, B). In 16G punctured discs, the AF underwent infolding accompanied by extensive scar tissue formation in the puncture lesion (Fig. 8C, D). Small blood vessels were seen to infiltrate the scar tissue and (at variable degrees) the outer AF, but they did not extend into the inner AF (Fig. 8c). In the AF defect discs, HE staining showed that the annular defect had lost its lamellar structure and was filled by extensive fibrocartilaginous-like tissue. Small fissure penetration into the repair tissue was typically observed at a limited depth (Fig. 8E). Invasion of blood vessels was also observed between the periannular muscle and the outer AF (Fig. 8e). Safranin-O staining revealed a rich proteoglycan content in the fibrocartilaginous tissue (Fig. 8F). In the PLGA/fibrin discs, the newly regenerated tissue formed a concave channel following the track of the AF defect and was well integrated with the inner part of the AF (Fig. 8G). There were also small residues of PLGA scaffold resulting from polymer degradation, surrounded by clusters of newly generated tissue (Fig. 8g). In addition, safranin-O staining revealed a severe reduction of proteoglycan content in the AF defect track, except for the boundaries of regenerated tissue and throughout the entire cartilaginous tissue (Fig. 8H).

### Immunohistochemistry

**Fig. 9:**
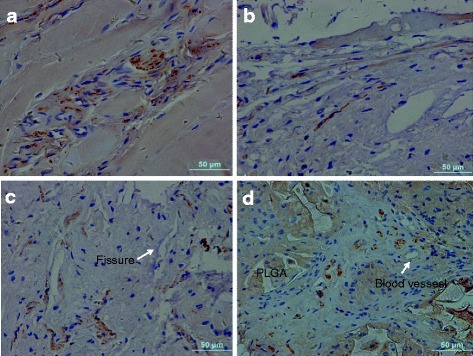
Immunohistochemical staining of PGP 9.5 at 6 months after surgery. The intact AF showed sparsely PGP 9.5-positive nerves distributed within the outermost layers of the AF (**a**). The punctured disc exhibited positive staining for PGP 9.5 scattered in superficial areas of the scar tissue (**b**). Small nerves were detected in the deeper outer AF along the fissures toward the NP (**c**). Nerves were seen deep within the inner AF, in close proximity to blood vessels (**d**)

PGP 9.5-positive nerves were sparsely distributed within the outermost layers of the AF, and the morphology of the nerves varied depending on the orientation of the nerves in the section (Fig. 9a). In 16G punctured discs, scar tissue was formed on the surface of the puncture sites. Positive staining for PGP 9.5 was scattered in superficial areas of the scar tissue (Fig. 9b). In the discs of the AF defect group, small nerves extended further into the deeper outer AF along the fissures toward the NP, but nerves were barely seen to invade the inner AF (Fig. 9c). In the PLGA/fibrin discs, in contrast, nerves extended into inner AF and were identified in the newly formed tissue surrounding the residual PLGA material, as well as deep within the inner AF. In some cases, but not always, nerves were seen in close proximity to blood vessels toward the NP (Fig. 9d).

**Fig. 10:**
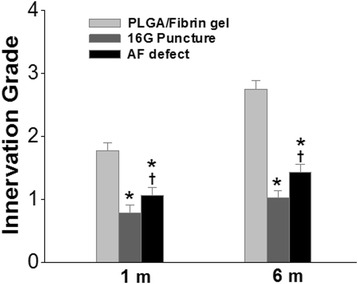
Changes in innervation scores at 6 months after surgery. The innervation score in the PLGA/fibrin gel implantation group was significantly higher than in the 16G puncture group or the AF defect group (1 and 6 months, vs. the 16G puncture group or annulus defect group,*P < 0.01). The innervation score of the AF defect discs was significantly higher than that of the 16G punctured discs. (^†^ *P* < 0.05 vs. the 16G puncture group)

In terms of semi-quantitative innervation scores, these increased slightly in all operated discs over the follow-up period. Notably, the PLGA/fibrin group showed a significantly higher innervation score compared with either the 16G puncture group or the AF defect group (1 and 6 months, *P* < 0.01). The innervation score of the AF defect discs was significantly higher than that of 16G punctured discs (*P* < 0.05, Fig. 10).

## Discussion

Animal models and lumbar specimens from humans with degenerated IVD show the presence of sensory nerve innervation, indicating a deep nerve invasion into the inner layer of the degenerated disc [40, 41]. In the classic annulus injury model, progressive degenerative changes are made to create partial- or full-thickness surgical lesions of the AF [42–44]. More recently, efforts have been made to examine the innervation of the injured disc in the annular rupture model [9, 10, 45, 46]; however, nerve ingrowth was seen to be confined within the scar tissue or outer third of the annulus, and no deeper annular or nuclear innervation was observed [9, 46]. We previously reported that, in a lumbar IVD injury model, annular injury produces progressive disc degeneration in rabbits [23]. In the present study, we examined the innervation of the degenerated discs. The results showed that this multi-segment model is well suited for the pre-clinical assessment of neuropathological changes induced by disc degeneration, as shown by radiographic imaging, MRI, real-time PCR, biochemical analysis, and histology/immunohistology.

In general, the intrinsic structure of normal discs is almost completely avascular and the innervation is limited to the outer layers of the AF [40, 47]. During disease states, this structure becomes disrupted, probably allowing the inappropriate entry of nerves and blood vessels, ultimately leading to pain generation. Several factors potentially influence nerve ingrowth into the normal IVD: (i) structural disruption (e.g., ingrowth of periannular innervated and vascularized granulation tissue along the fissures [12, 46]), (ii) regulators of innervation (e.g., increase of inflammatory mediators, neurotrophins, and angiogenic factors [48–50]), (iii) increase of matrix disorganization (repulsive factors, e.g., aggrecan, which inhibits nerve fiber growth in vivo/in vitro [13, 19, 51]), and (iv) abnormal mechanical stress [52, 53].

Thus far, annular injury is the most common surgical modality in various animal models of disc degeneration [44]. A potential concern of annulus injury models is that surgical structural disruption in the annulus wall using needles or drill may result in the leakage of nuclear contents. Less invasive annulotomy for rabbit models of IVD degeneration, as chosen in the present study, has been devised as a slow and moderate model of disc degeneration. Nonetheless, the risk of early leakage of the nuclear contents through the defects generated by the annulotomy itself cannot be excluded. The approach of the present study appeared to induce a slower, more controlled leakage of the nuclear contents, in line with previous needle puncture models with needles of various sizes [9, 54, 55]. Furthermore, noticeable scar tissue formed in the needle track and lacked PGP 9.5-positive nerve fibers further into the AF, only shown in superficial areas.

Theoretically, a relatively minor structural disruption was generated in the punctured annulus in contrast to AF defect in the annulotomy-induced disc. Since experimental ovine annular lesions displayed a spontaneous repair in the outer AF [22], the present study sought to minimize the potential effect of localized annulus seal, i.e., AF defects were created by drilling, allowing nerves and vascular access from the peripheral disc to penetrate the outer AF. Finally, the AF defect sites showed that observable fibrocartilaginous tissue was formed in the annular wall with typical fissures. Moreover, there was evidence of more extensive nerves penetration along the fissures, as indicated in Fig. 9c. Also, PGP 9.5-positive nerves invaded the deeper part of the AF, and small blood vessels invaded the boundary between the periannular muscle and the outer AF. Nevertheless, the innervation degree of the AF defect was significantly higher than that seen with the 16G puncture, indicating a slight advantage of the empty defect for the nerve ingrowth via AF defect. Therefore, unsealing of the annulus wall appears to facilitate a deeper nerve penetration; in particular, empty defect discs and annulus fissures are associated with the ingrowth of nerves, in agreement with previous results [12, 13, 46, 48].

Recently, several tissue-engineering studies have focused on the quality of repair tissue in comparison to native discs, particularly in view of replacement of the AF [56–60]. Interestingly, however, there is still limited information on the effects of nerve ingrowth into the defect or the implanted site during IVD degeneration. In the present study, implantation of PLGA/fibrin gel constructs appeared effective in inducing a slower disc degeneration and in promoting the full integration of the regenerated tissue, both aspects essentially contributing to the longevity of the repair [29, 61, 62]. At the same time, the resulting repair promoted the growth of peripheral PGP 9.5-positive nerves and of a few blood vessels into the deeper parts of the injured disc (Fig. 9d). In addition, discs sealed with PLGA/fibrin gel showed a significantly higher innervation score compared to 16G punctured or empty defect discs. In particular, the innervation was found in close proximity to small blood vessels in the regenerated tissues, in agreement with previous investigations [5, 10, 11, 63]. To our understanding, the ingrowing nerves need to be supplied by blood vessels. Also, vascular proliferation in regenerated tissue essentially contributed to full degradation of polymeric constructs and natural shrinking of the extruded NP in the early stage repair. However, the relatively short observation period of the present study did not allow a detailed histological analysis of the innervation changes.

The presence of aggrecan in the disc may play a key role in inhibiting the extension of nerve fibers [10, 13, 19]. In the present biochemical analysis, the injured AF structure showed a significant reduction of the proteoglycan content in comparison to normal discs. Filling the defects with PLGA/fibrin gel constructs, however, resulted in even greater proteoglycan loss than in unsealed AF defects or annular puncture. Notably, the proteoglycan loss was particularly pronounced in the AF channel of implanted site, as indicated by safranin-O staining. NP extrusion did not reduce the significant loss of proteoglycan content, possibly due to scar tissue surrounding the NP in the AF puncture track, as shown in previous studies [21, 64]. In the present study, extensive fibrocartilage-like tissue with pronounced proteoglycan content was observed in the AF defect or puncture sites, with possible inhibitory effect on nerve ingrowth into the inner layers of the AF and NP. As a result, early leakage of the NP with depletion of proteoglycan may facilitate nerve ingrowth into the deeper AF in the rabbit annular injury degeneration model.

### Limitation

PGP 9.5 is a highly specific marker of nerve tissue with a limited background staining [65, 66]. However, the presence of sensory nerve fibers more specifically related to pain sensation was not further specified in the present study. Also, the “insert” scaffolds may have unfavorable effects on the innervated and vascularized environments. In addition, given a significant anatomic and biomechanical difference in the rabbit spine compared with the human spine, the neuropathological and degenerative changes observed in the rabbits may not be representative of possible effects in humans. Thus, a long-term evaluation of the pathological changes of innervated and vascularized discs is warranted in a larger animal model of IVD.

## Conclusions

A new rabbit disc degeneration model with drilling of a defect in the AF appears to be useful for the evaluation of nerve and/or blood vessel ingrowth in the degenerated discs. Implantation of PLGA/fibrin gel showed a significantly higher nerve invasion compared to empty defect and annulus puncture. Possible factors underlying this difference include (i) more nerves grow into the AF defect along the fissures, (ii) biodegradable PLGA/fibrin gel products may support the regeneration of the AF and subsequently promote the growth of peripheral nerves and blood vessels into the deeper parts of the injured disc, and (iii) ingrowth may be facilitated by early loss of proteoglycan.

## Abbreviations

AF: Annulus fibrosus
ANOVA: Analysis of Variance
DHI: Disc height index
DMMB: 1,9-Dimethylmethylene blue
EDTA: Ethylenediamine tetraacetic acid
GAG: Glycosaminoglycans
GAPDH: Glyceraldehyde-3-phosphate dehydrogenase
HE: Hematoxylin/eosin
HRP: Horseradish peroxidase
IVD: Intravertebral disc degeneration
LSD: Least Significant Difference
MRI: Magnetic resonance imaging
NP: Nucleus pulposus
PACS: Picture archiving and communication system
PCR: Polymerase chain reaction
PLGA: Poly(lactic-co-glycolic acid)
